# The effect of biological sex on cool seeking behavior during passive heat stress in young adults

**DOI:** 10.1007/s00421-025-05702-8

**Published:** 2025-02-13

**Authors:** Hui Wang, Zachary J. Schlader, Tze-Huan Lei, Toby Mündel, Tatsuro Amano, Naoto Fujii, Takeshi Nishiyasu, Narihiko Kondo

**Affiliations:** 1 Laboratory for Applied Human Physiology, Graduate School of Human Development and Environment, Kobe University, 3-11 Tsurukabuto, Nada-Ku, Kobe, 657-8501 Japan; 2 Department of Kinesiology, Indiana University School of Public Health, Bloomington, IN USA; 3 College of Physical Education, Hubei Normal University, Huangshi, China; 4 Department of Kinesiology, Brock University, St. Catharines, ON Canada; 5 Faculty of Education, Niigata University, Niigata, Japan; 6 Institute of Health and Sports Science, University of Tsukuba, Tsukuba, Japan

**Keywords:** Sex difference, Cool seeking behavior, Perceptual response, Passive heat stress, External cooling

## Abstract

**Purpose:**

This study tested the hypothesis that females engage in cool seeking behavior to a greater extent during passive heating compared to males.

**Methods:**

27 healthy participants (14 males) underwent two trials of 50 min lower leg passive heating with (Fan trial) and without the fan (No fan trial) in a 27 °C, 50% relative humidity environment. In the Fan trials, participants were allowed to use the fan by pressing the button to keep themselves comfortable while they were not allowed in the No fan trial.

**Results:**

Cool seeking behavior was initiated at the same change (∆) in rectal temperature (0.2 (0.2) °C vs 0.2 (0.1) °C, *p* = 0.281) and ∆ mean skin temperature (2.1 (0.6) °C vs 2.3 (0.6) °C, *p* = 0.307), but cooling time was longer (13.5 (5.4) min vs 17.3 (3.9) min, *p* = 0.040) and cumulative number of times pressing the button is more often (7.3 (3.6) times vs 10.8 (4.6) times, *p* = 0.049) in females compared to males. Thermal sensation, thermal discomfort, and perceived skin wetness were not different between sexes during lower leg passive heating in all trials (all *p* > 0.145). Furthermore, whole body sweat loss and local sweat rate on the forearm were significantly higher in males compared to females (all *p* < 0.042) across Fan and No fan trials.

**Conclusion:**

In conclusion, females engage in cool seeking behavior to a greater extent than males. Furthermore, thermal perceptions are not different between sexes during passive heating.

## Introduction

Behavioral thermoregulation is the most powerful and diverse thermoregulatory response (Schlader and Vargas 2019). Cool seeking behaviors are initiated upon the sensation of thermal discomfort, secondary to an increase in body temperatures (Bulcao et al. 2000; Schlader et al. 2011) and skin wetness (Vargas et al. 2020). It is reported that females are more perceptually sensitive than males to a variety of stimuli, including pain and heat (Feine et al. 1991; Lautenbacher and Strian 1991; Averbeck et al. 2017; Golja et al. 2003; Gerrett et al. 2014). It is speculated that these differences may be due to a greater density of thermoreceptors per unit body surface area (Kanosue et al. 2010). Such sex-dependent differences likely explain the observation that during exercise in a moderate thermal environment, females engage in cool seeking behavior to a greater extent than males (Vargas et al. 2019b).

Observations of sex related differences in cool seeking behavior during exercise (Vargas et al. 2019b) may not always translate to that occurring during passive heat stress at rest. Thus, the passive heat stress was applied in this study to offer a controlled and isolated environment to study core thermoregulatory mechanisms, which allows us to distinguish between metabolic heat production (as seen in exercise) and externally applied heat. Also, exercise-induced analgesia often blunts the thermal perception of both noxious and innocuous thermal stimuli (Koltyn 2000; Ouzzahra et al. 2012; Gerrett et al. 2014), likely due to attenuated transmission of sensory information to the thalamus and somatosensory cortex induced by movement (Coulter 1974). Moreover, the threshold for detecting cutaneous stimuli is increased during active movement (Garland and Angel 1974) likely because movement reduces or interrupts sensory information flow and central nervous system integration (Rushton et al. 1981). Furthermore, it has been proposed that exercise-induced stress hormones probably play a key role in the reduction of thermal stimuli sensitivity (Janal et al. 1984; Kemppainen et al. 1985). Given these effects of exercise on thermal perception compared with rest, whether the utilization of cool seeking behavior differs between males and females during passive heating warrants further investigation. This study could provide insight into how the body copes with ambient heat stress independently of physical activity, and enacting effective mitigation strategies for occupations, especially females to prevent them from heat related illness. (Carter et al. 2005; Druyan et al. 2012; Kazman et al. 2015).

With this background, the purpose of this study was to test the hypothesis that engagement in cool seeking behavior would be greater in females compared to males using a model of passive heat stress.

## Methods

### Participants

27 healthy young adults (14 males) were recruited for this study. Based on our pilot data and previous data (Vargas et al. 2019b) that investigated the effect of sex on behavioral thermoregulation, at an alpha level of 0.05 and an effect size (*f*) of 0.33, a minimum sample size of 10 participants for each group is required and would result in a power of 0.96 that would be sufficient to detect a significant between-within subject interaction. Participants' physical characteristics are summarized in Table 1. All participants were aware of the study's equipment and approach and did not take any medications. None of the participants had any known neurological, metabolic, cardiovascular, or mental illnesses. All females were tested within the early/mid-follicular phase, defined as the first 10 days from the onset of menstruation, to control for the influence of female sex hormones (Vargas et al. 2019b).

**Table 1:** Physical characteristics for male (n = 14) and female (n = 13) participants

Group	Age (years)	Height (cm)	Mass (kg)	Bodyfat (%)	BSA (m^2^)	VO^2^_peak_ (mL kg^−1^ min^−1^)
Male	23.9 (3.2)	174.7 (6.5)	63.3 (8.9)	16.0 (4.1)	1.72 (0.33)	43.4 (7.9)
Female	24.6 (3.7)	159.1 (4.0)	53.3 (5.3)	28.5 (2.7)	1.50 (0.27)	31.3 (4.7)
*p*-value	0.613	0.001*	0.002*	0.000*	0.001*	0.000*

*Indicates a significant difference between males and females, *p* < 0.05, determined by t-test

### Experimental overview

Participants were asked to visit the laboratory on three separate occasions. Visit 1 was a screening and familiarization visit while visits 2 and 3 were the experimental trials. The experimental trials involved lower leg passive heating with and without an electric fan. All trials were performed in a fixed order (without an electric fan and then with an electric fan) to ensure that everyone had the same previous experience with the heating. Each trial was separated by at least 48 h and was conducted in the morning between 8 and 11 AM to minimize the effect of circadian rhythm on body temperature fluctuation, and 2 h postprandial. All trials were conducted during the middle of January to the end of February in Kobe, Japan where the average highest ambient temperature was below 10 °C to avoid heat adaptation by seasonal acclimatization.

### Experimental trials

**Fig. 1:**
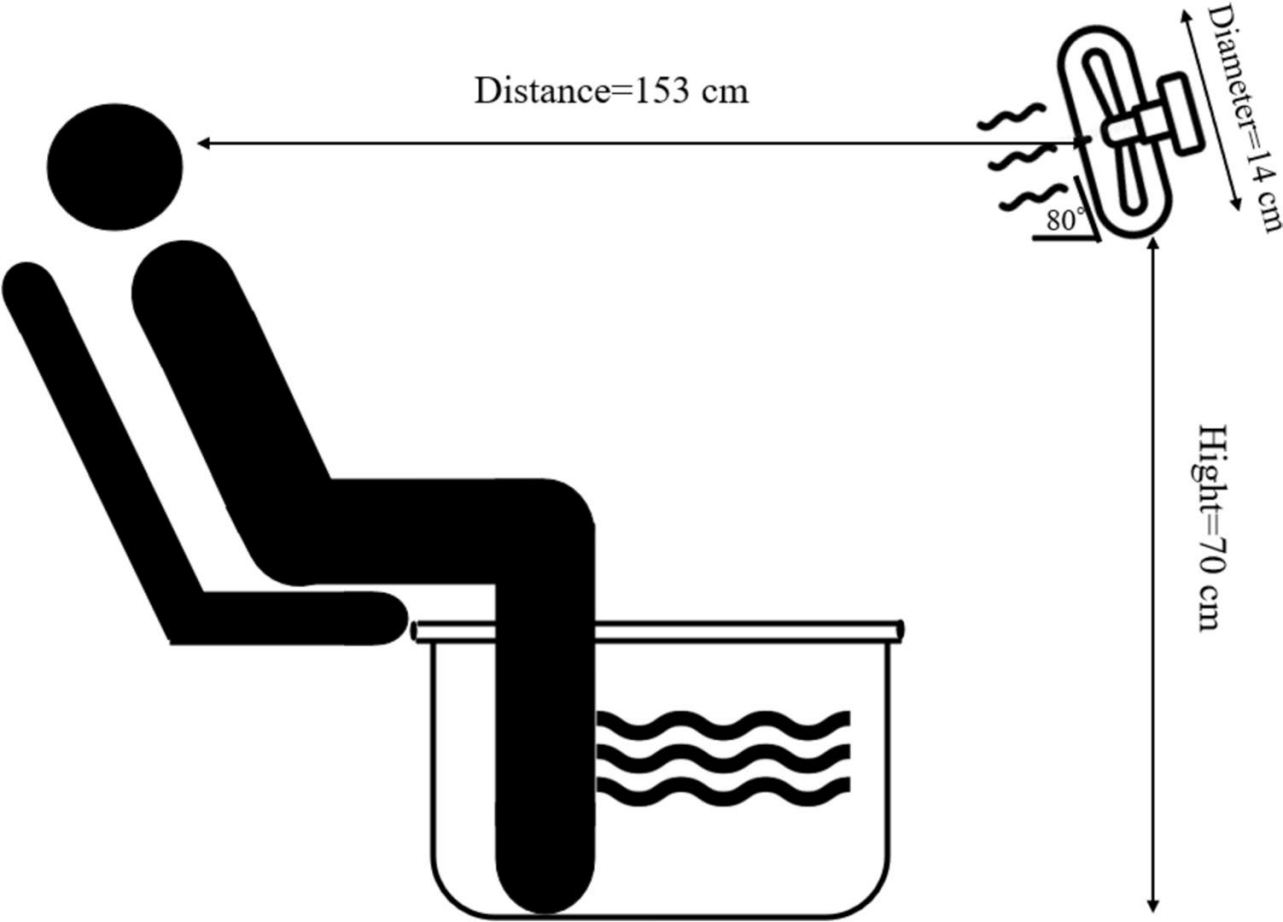
The schematic figure for the position of the fan

Before entering the chamber, participants provided a urine sample for the assessment of hydration status. Euhydration was confirmed by urine-specific gravity with no participants exceeding the value of 1.015 (Cheuvront and Sawka 2005). Nude body weight was measured using high-precision weight scales and a rectal thermometer was then self-inserted 10–12 cm beyond the anal sphincter. All participants in the experimental trials wore shorts (and a sports bra for females) in the climatic chamber set to 27 °C and 50% relative humidity. Each trial was 85 min in duration, which consisted of a 5-min baseline, a 50-min lower legs immersion in the semi-supine position, and a 30-min skin blood flow (SkBF) max procedure (the legs were out of the water during SkBF max test). Subjects were not allowed to drink in any trials. Figure 1 depicts the experimental setup involving the fan placement and lower leg hot water immersion while in the semi-supine position. In the Fan trial, participants were allowed to behaviorally thermoregulate by using the fan as often as they desired during lower leg passive heating. When the subject pressed the button, the fan turned on for two minutes after which a mandatory one-minute wash-out phase commenced. The use of the washout was necessary to ensure a continual drive to seek cooling. The airflow of the fan did not differ between males and females (0.69 (0.07) m/s in males vs 0.66 (0.09) m/s in females, *p* = 0.53). The special switch for controlling the fan (MaP3058ASCD; Nihon Santec, Osaka, Japan) was the same as our previous study (Wang et al. 2023). Thermal perceptions were measured at every 10-min interval in both trials.

### VO_2 peak_ measurement and lower leg passive heating

On visit 1, each participant underwent a VO_2 peak_ test by ergometer with a workload that began at 20 W for the first two minutes and increased by 15 W every minute for females and 30 W/min for males. Subjects were instructed to maintain a cadence of 60 rpm until volitional exhaustion. The criteria for VO_2 peak_ were the following if three of the five conditions were met: a respiratory exchange ratio ≥ 1.10, a plateau in VO_2_ with increasing workloads, workload volitional fatigue (< 55 revolutions per minute cadence), exercise heart rate that was within 10 beats of the age-predicted maximal heart rate ([220-age] × 0.95), rating of perceived exertion (RPE) of 19–20 (Rivas et al. 2020).

The passive heat stress protocol began with submerging the legs up to the knees in a 42 °C stirred water bath (Kuwahara et al. 2005a; Inoue et al. 2005a; Kuwahara et al. 2005b; Heinonen et al. 2011; Crandall and Wilson 2015) while the participants were in a semi-supine position. This moderate level of heat exposure allowed for gradual and progressive elevations in core temperature (T_c_), thus allowing examination of changes in the time course of autonomic and behavioral thermoeffector activation. Moreover, this approach has less central fluid displacement, and more realistic mean skin temperatures in the non-immersed areas compared to whole-body immersion or heating with a water-perfused suit (Crandall and Wilson 2015). Additionally, it has been reported that changes in core temperature strongly contribute to the initiation of thermoregulatory behavior in hyperthermic conditions (Vargas et al. 2019a), and lower leg passive heating allowed more internal heat strain than external (i.e., cutaneous) heat strain in this study.

### Measurements

*Physical characteristics*: Height and body mass were measured using a stadiometer (Seca, Germany; accurate to 0.1 cm) and scale (Mettler-Toledo, Germany; accurate to 10 g). The percentage of body fat was determined by Inner skin (4C Technology, TANITA Corporation, Japan). Body surface area (BSA) for each subject was calculated from their height and mass (Fujimoto and Watanabe 1969), which were measured using standard techniques: BSA = body mass (kg)^0.444^ × height (cm)^0.663^ × 0.008883.

*Airflow:* The airflow of the fan was measured by an anemometer (KANOMAX, Japan). The mean value was calculated by the airflow of four body sites (forearm, abdomen, chest, and neck).

*Cardiovascular parameters:* Heart rate was recorded from the detection of R-R intervals (Polar Vantage XI, Polar Electro, Finland) whilst blood pressure was measured continuously using a Finometer device (Finapres Medical Systems, Amsterdam, The Netherlands) throughout experimental sessions.

Skin blood flow (SkBF) was measured on the left mid-medial forearm (SkBF_forearm_) and left upper chest (SkBF_chest_) using laser-Doppler velocimetry (ALF21; Advanced, Tokyo, Japan). A 3.8 cm-diameter hollow circle heater element with an integrated laser Doppler flow probe inside the hollow circle was used to standardize SkBF max to induce the maximal amount of cutaneous vasodilation, a skin temperature sensor was placed close to the heater to monitor the local skin temperature response until a local skin temperature of 42°C was obtained (Cui et al. 2006). Unfortunately, blood pressure could not be measured during local heating. Thus, we reported SkBF as an index of cutaneous vasomotor activity given that blood pressure did not differ throughout the two conditions, which is similar to procedures utilized in our previous study (Lei et al. 2021). All SkBF values during lower leg passive heating were converted to percentages of the SkBFmax value [%SkBF = (SkBF/SkBF max) × 100].

*Core and skin temperatures:* Rectal temperature (T_re_) and local skin temperature on the thigh (T_thigh_), calf (T_calf_), chest (T_chest_), and forearm (T_forearm_) were measured continuously by using copper-constantan thermocouples (Inui Engineering, Higashi Osaka, Japan) and secured using surgical tape (3 M Micropore). A rectal thermocouple (Physitemp, RET-1, USA) was inserted 10–12 cm beyond the anal sphincter to measure T_re_. Mean skin temperature (T_sk_) was calculated according to the equation of Ramanathan (Ramanathan 1964). T_re_ and local skin temperature were recorded by a data logger with a sampling rate of 1 Hz and displayed on the monitor continuously. Data were expressed as a 5-min average until the end of the trial. In addition, mean body temperature (T_b_) was calculated with 0.8T_re_ + 0.2T_sk_ (Hardy and Stolwijk 1966).

*Sudomotor function measurements:* The ventilated sweat capsule technique was used to measure the local sweat rate (LSR) on the left mid-medial forearm (LSR_forearm_) and left upper chest (LSR_chest_). Before each experiment, dry nitrogen gas was pumped (500 ml/min) through the apparatus for around an hour to ensure that the relative humidity level was closer to zero percent. At least 30 min before data collection, collodion glue was used to attach each capsule (3.46 cm^2^) to the skin. A capacitance hygrometer (HMP50; Vaisala, Helsinki, Finland) was used to measure the temperature and humidity of the efferent gas. Using high-precision weight scales (Mettler-Toledo, B60, Germany), the participant's nude body mass was measured before and after lower leg passive heating for the assessment of whole-body sweat loss.

*Behavioral thermoregulation:* The primary thermoregulatory behavior assessments were the time when the 1st behavior occurred (1st time the fan turned on), the cumulative number of times turned on the fan, the cumulative times of pressing the button (including pressing the button during one-minute wash out phase), the total amount of time individuals spent receiving cooling and the slope of the cumulative times of pressing button relative to the change (∆) in T_b_, all of which offered quantitative measurements of cool-seeking behavior (Vargas et al. 2020). T_re_, T_b_, T_sk_, and their increased magnitude when the 1st behavior was initiated were also recorded.

*Thermal perceptions:* Whole body thermal perception used the following standard scales: thermal sensation (− 3 = very cold, − 2 = cold, − 1 = cool, 0 = neutral; + 1 = warm, + 2 = hot, + 3 = very hot), thermal discomfort (− 3 = very uncomfortable, − 2 = uncomfortable, − 1 = slightly uncomfortable, 0 = neutral; + 1 = slightly comfortable, + 2 = comfortable, + 3 = very comfortable) and wetness (− 3 = very wet, − 2 = wet, − 1 = slightly wet, 0 = neutral; + 1 = slightly dry, + 2 = dry, + 3 = very dry) (Olesen and Brager 2004).

### Data and statistical analyses

To determine the onset of LSR in the forearm and chest region against T_b_, we employed 1-minute averages. The LSR was plotted against the incremental T_b_ from baseline (∆T_b_) during lower leg passive heating. The onset of the sweating against ∆T_b_ was determined by substantial deviation from the baseline values using segmental regression according to Cheuvront (Cheuvront et al. 2009). The slope of the regression line between the point of onset and before the plateau was used to determine the thermal sensitivity of the sweating response. We also evaluated the slope of the cumulative times of pressing the button for fan use plotted against T_b_ as the responsiveness of cool-seeking behavior to increases in body temperature in two groups.

Resting autonomic, cardiovascular data and behavioral parameters were compared between males and females using an unpaired sample t-test. Consistent with our hypothesis, the effect of sex difference on cardiovascular variables, body temperature, sweating response, skin blood flow, and thermal perceptionduring passive heat stress was examined using a 2-way mixed model ANOVA (sex × time) in the Fan trials only. To ensure that any observed effects of sex in the "Fan" condition can be attributed specifically to the use of the fan, and provide a necessary baseline for comparing the physiological and behavioral responses observed in the "Fan" condition, we also examined the sex difference in perceptual and autonomic responses during passive heat stress during a situation in which cool seeking behavior was prohibited using a 2-way mixed model ANOVA (sex × time) in the No fan trials only. By design, comparisons between Fan and No fan trials were not conducted, given that the physiological effect of voluntary fan use under similar circumstances has been reported previously (Wang et al. 2023). When the outcome of an ANOVA revealed a statistical interaction, post hoc comparisons were made using Sidak-adjusted comparisons. Statistical significance was set at *p* ≤ 0.05. Descriptive statistics were presented as mean (SD), and all variables were reported as mean $\pm$ SD at 95% confidence intervals. The normality of the data was examined by the Kolmogorov–Smirnov Test. All data were processed by GraphPad Prism software (Prism, version 8, San Diego, USA).

## Results

### Physical characteristics

Males and females did not differ in age (*p* = 0.613). Females were shorter (*p* = 0.001), had smaller body mass (*p* = 0.002), and lower BSA (*p* = 0.001) compared to males, but had greater percent body fat (*p* < 0.001), and had a lower VO_2peak_ (*p* < 0.001) (Table 1).

### The effect of sex difference on cool seeking behavior during passive heating

#### Cool-seeking behavior parameters

The time of 1st turn on the fan and the cumulative time of turning on the fan did not differ between the two groups (all *p* > 0.345, Table 2). Similarly, ∆T_re_, ∆T_sk_, and ∆T_b_ upon fan initiation did not differ between males and females (all *p* > 0.071, Table 2). However, the total cooling time and cumulative time of pressing the button was higher in females compared to males (all *p* < 0.049, Table 2). Furthermore, the slope of the relationship between cumulative times of pressing the button and rising T_b_ was significantly higher in females compared to males (26.00 (11.43) times/ °C vs 17.03 (8.16) times/ °C, *p* = 0.043, Table 2).

**Table 2:** Behavioral parameters in both groups in Fan trials, N = 27, male (n = 14) and female (n = 13)

	Time to first behavior (min)	Total cooling time (min)	Cumulative number of time turning the fan (times)	Cumulative number of times pressing the button (times)	∆T_re_ when 1st turn on the fan (°C)	∆T_sk_ when 1st turn on the fan (°C)	∆T_b_ when 1st turn on the fan	Slope of thermal behavior (presses/°C)
Male	19.7 (6.7)	13.5 (5.4)	6.3 (4.4)	7.3 (3.6)	0.2 (0.2)	2.1 (0.6)	0.6 (0.1)	17.0 (8.2)
Female	19.0 (6.7)	17.3 (3.9)	7.9 (2.4)	10.8 (4.6)	0.2 (0.1)	2.3 (0.6)	0.7 (0.2)	26.0 (11.4)
*p*-value	0.782	0.040*	0.345	0.049*	0.281	0.307	0.071	0.043*

*Indicates a significant difference between males and females, *p*-values are reported, determined by t-test

#### Cardiovascular variables

No sex differences in heart rate were observed in the Fan trials (Average value: 78 (6.2) beats/min vs 76 (5.7) beats/min, *p* = 0.576) during lower leg passive heating. Moreover, MAP was not different between males and females throughout the protocol (Average value: 75 (1.6) mmHg vs 80 (1.4) mmHg, *p* = 0.253). SBP and DBP were also not significantly different between the groups (all *p* > 0.116).

#### Body temperature

**Fig. 2:**
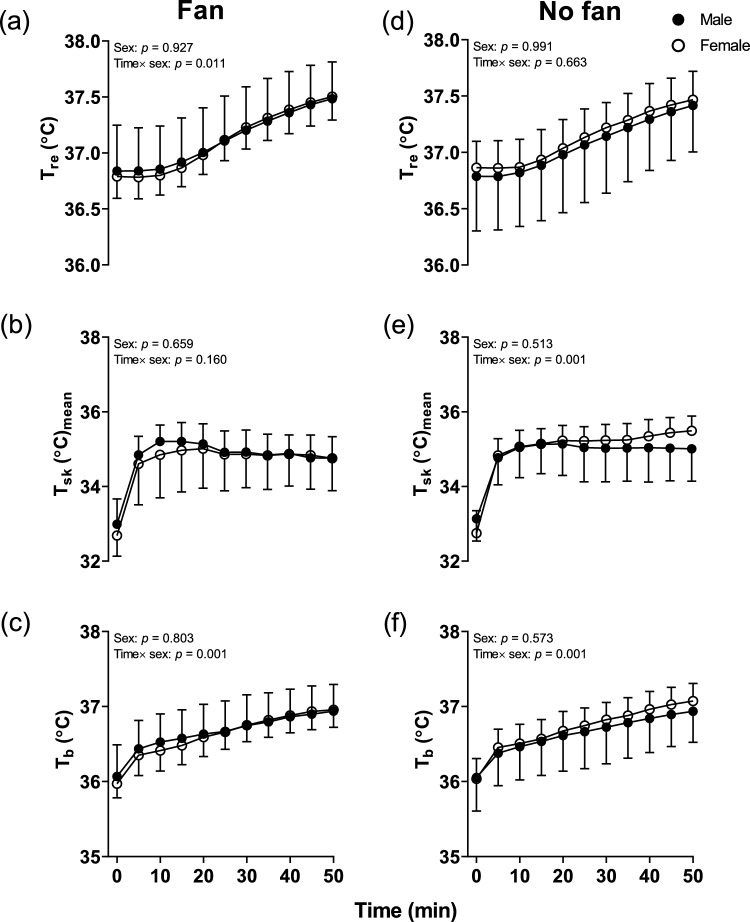
Time course of changes in rectal temperature (T_re_), mean skin temperature (T_sk_) and mean body temperature (T_b_) during lower leg immersion between males and females in Fan and No fan trials. N = 27 (male = 14, female = 13), solid circles are males whilst open circles indicate females. * Indicates a significant difference between the male and female groups at individual time points from Sidak post hoc testing. During lower leg passive heating were analyzed by two-way mixed model ANOVA

The absolute values of T_re_, T_sk_, and T_b_ at baseline were not different between the two groups in the Fan trials (all *p* > 0.727). There was also no main effect of sex difference in T_re_ (Average value: 37.11 (0.28) °C vs 37.12 (0.25) °C, *p* = 0.927, Fig. 2a) throughout lower leg passive heating, it showed an interaction effect (Time × sex) (*p* = 0.011) even though we did not observe sex difference in all the time points from Post hoc testing (all *p* > 0.133, Fig. 2a).

T_sk_ also showed the same trend between sexes across lower leg passive heating in the Fan trials (Average value: 34.65 (0.66) °C vs 34.77 (0.61) °C, *p* = 0.660, Fig. 2b), we did not observe a main effect and interaction effect (all *p* > 0.160) in T_sk_. Moreover, there was no main effect of sex difference for T_b_ between males and females during lower leg passive heating (Average value: 36.62 (0.30) °C vs 36.65 (0.25) °C, *p* = 0.803, Fig. 2c), it showed an interaction effect (Time × sex) (*p* = 0.001) while we did not observe sex differences in all the time points from Post hoc testing (all *p* > 0.219, Fig. 2c).

#### Sweating response and skin blood flow

**Fig. 3:**
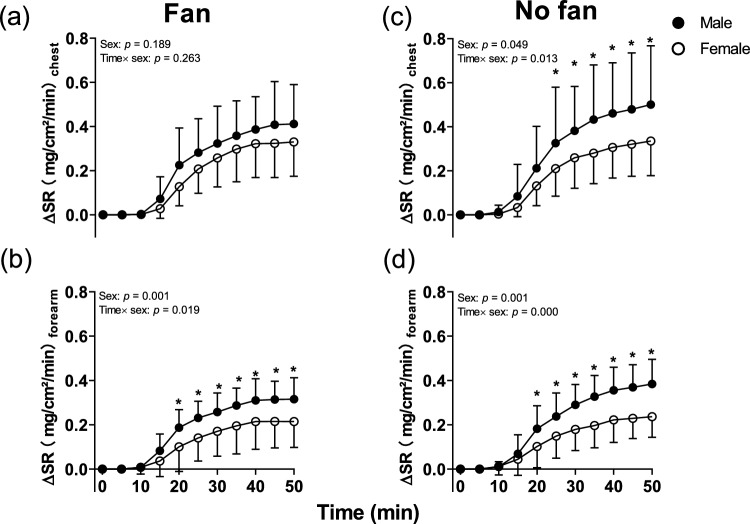
Time course of ΔLSR on the forearm and chest during lower leg immersion between males and females in Fan and No fan trials. N = 27 (male = 14, female = 13), solid circles are males whilst open circles indicate females. * Indicates a significant difference between the male and female groups at individual time points from Sidak post hoc testing. During lower leg passive heating were analyzed by two-way mixed model ANOVA

Males showed a greater ∆LSR at the forearm during passive heating in Fan trials from 20 min of passive heating (Average value: 0.18 (0.13) mg/cm^2^/min vs 0.12 (0.09) mg/cm^2^/min, *p* = 0.003, Fig. 3b). However, there was no main and interaction effect (Time × sex) on the chest in Fan trials (Average value: 0.22 (0.17) mg/cm^2^/min vs 0.17 (0.14) mg/cm^2^/min, all *p* > 0.189, Fig. 3a). When expressing LSR_chest_ and LSR_forearm_ relative to ∆T_b_, the onset of LSR is not different between sexes in Fan trials. Similarly, the sensitivity (slope) on the forearm and chest did not reveal a difference between the two groups (all *p* > 0.170, Table 3). However, whole body sweat loss was significantly higher in males compared to females in the Fan trials (0.32 (0.15) kg vs 0.23 (0.04) kg, *p* = 0.042).

**Table 3:** The onset threshold for and thermosensitivity of local sweat rate against mean body temperature (T_b_) at each site of the body (N = 27) in males and females across two trials

	Fan			No fan				
	Means (SD)		*p* value	Means (SD)			*p* value	
	Male	Female	Sex	Male		Female	Sex	
Onset T_b_ (℃)								
Chest	36.57 (0.41)	36.59 (0.33)	0.911	36.50 (0.42)		36.66 (0.22)	0.288	
Forearm	36.55 (0.40)	36.59 (0.34)	0.764	36.50 (0.41)		36.65 (0.22)	0.264	
Slope (mg/cm^2^/min/°C)								
Chest	0.78 (0.25)	0.70 (0.30)	0.503	1.05 (0.54)		0.78 (0.25)	0.170	
Forearm	0.65 (0.40)	0.45 (0.24)	0.168	0.92 (0.45)		0.53 (0.26)	0.024*	

Significantly differ between males and females, *p*-values are reported, determined by t-test

**Fig. 4:**
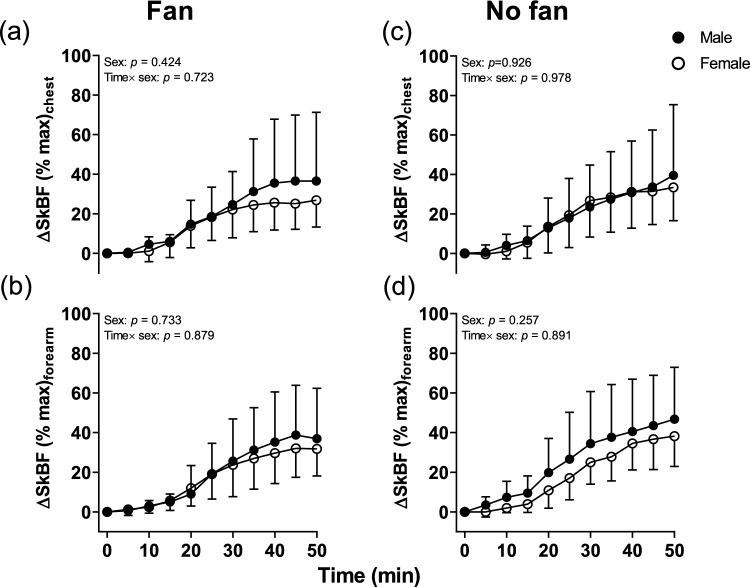
Time course of Δ SkBF (%max) on the forearm and chest during lower leg immersion between males and females in Fan and No fan trials. N = 27 (male = 14, female = 13), solid circles are males whilst open circles indicate females. * Indicates a significant difference between the male and female groups at individual time points from Sidak post hoc testing. During lower leg passive heating were analyzed by two-way mixed model ANOVA

Furthermore, ΔSkBF on the forearm and chest did not reveal a significant main effect for sex or an interaction effect (Time × sex) for the sex in Fan trials (all *p* > 0.424, Fig. 4a and b).

#### Thermal perceptions

**Fig. 5:**
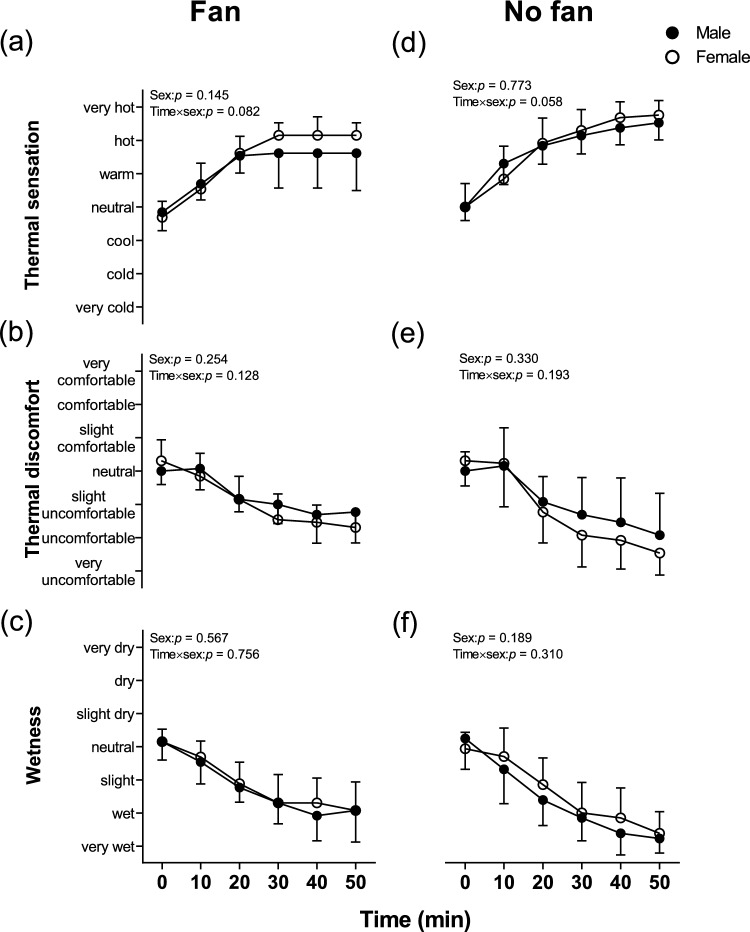
Time course of thermal perception changes during lower leg immersion between males and females in Fan and No fan trials. N = 27 (male = 14, female = 13), solid circles are males whilst open circles indicate females. * Indicates a significant difference between the male and female groups at individual time points from Sidak post hoc testing. During lower leg passive heating were analyzed by two-way mixed model ANOVA

Thermal sensation, thermal discomfort, and wetness perception did not differ at baseline between the groups (all *p* > 0.597, Fig. 5). Thermal sensation did not reveal any statistically significant difference (main effect and interaction effect) between males and females (Average value: 1.39 (1.04) vs 1.15 (0.74), *p* = 0.15, and *p* = 0.082, Fig. 5a). Moreover, there was no main effect of sex difference and interaction effect (Time × sex) in thermal discomfort (Average value: − 0.72 (0.43) vs -0.89 (0.34), *p* = 0.254, Fig. 5b), and wetness perception (Average value: -1.21 (0.47) vs − 1.03 (0.46), *p* = 0.567, Fig. 5c) in the Fan trials.

### The effect of sex difference on perceptual and autonomic responses during passive heat stress (When cool-seeking behaviour was restricted)

#### Cardiovascular variables

No sex differences in heart rate were observed in the No fan trials (Average value: 81 (6.5) beats/min vs 75 (5.2) beats/min, *p* = 0.176) during lower leg passive heating. Moreover, MAP did not differ between males and females throughout passive heating (Average value: 79 (0.9) mmHg vs 75 (1.8) mmHg, *p* = 0.440). SBP and DBP were also not significantly different between the groups (all *p* > 0.153).

#### Body temperature

The absolute values of T_re_, T_sk_, and T_b_ at baseline were not different between the two groups in the No fan trials (all *p* > 0.327). There was no main effect of sex difference and interaction effect (Time × sex) in T_re_ in the No fan trials (Average value: 37.13 (0.23) °C vs 37.07 (0.23) °C, *p* = 0.991 and *p* = 0.663 in No fan, Fig. 2d) throughout lower leg passive heating.

T_sk_ also showed the same tendency between sexes across whole lower leg passive heating (Average value: 35.00 (0.76) °C vs 34.85 (0.58) °C, *p* = 0.513 in No fan, Fig. 2e). There was no main effect of sex difference in T_b_ during lower leg passive heating (Average value: 36.70 (0.30) °C vs 36.63 (0.26) °C, *p* = 0.573 in No fan, Fig. 2f). Besides, the T_sk_ and T_b_ in No fan trials showed an interaction effect (sex × time) (all *p* < 0.001) whilst we did not observe seasonal difference in all the time points from Post hoc testing (all *P* > 0.093, Fig. 2e and f). Despite the significant interaction, these results suggest that while the overall time-related trends between sexes differed, there were no substantial differences at specific moments during the immersion.

#### Sweating response and Skin blood flow

The males showed a greater ∆LSR at the forearm during passive heating in the No fan trials from 20 min of passive heating (Average value: 0.20 (0.16) mg/cm^2^/min vs 0.12 (0.10) mg/cm^2^/min, *p* = 0.001, Fig. 3d). Also, the main and interaction effect (Average value: 0.26 (0.20) mg/cm^2^/min vs 0.17 (0.14) mg/cm^2^/min, all *p* < 0.049) of ∆LSR on the chest was observed in No fan trials, post hoc test revealed the sex difference from 25 min of passive heating (Fig. 3c).

When expressing LSR_chest_ and LSR_forearm_ relative to ∆T_b_, the onset of LSR did not differ between sexes across two trials (all *p* > 0.264, Table 3). However, the sensitivity on the forearm was higher in males compared to females in No fan trials (Average value: 0.92 (0.45) mg/cm2/min/°C vs 0.53 (0.26) mg/cm2/min/°C, *p* = 0.024, Table 3). Whole body sweat loss was significantly higher in males compared to females in the No fan trials (0.36 (0.12) kg vs 0.26 (0.07) kg, *p* = 0.013).

ΔSkBF on the forearm and chest did not reveal a significant main and interaction effect for the sex in the No fan trials (all *p* > 0.257, Fig. 4).

#### Thermal perceptions

Thermal sensation, thermal discomfort, and wetness perception did not differ at baseline between two groups (all *p* > 0.724, Fig. 5). Furthermore, thermal sensation did not differ between males and females in the No fan trials, no main effect of sex difference and interaction effect (Time × sex) was observed (Average value: 1.71 (0.35) vs 1.76 (0.51), *p* = 0.773, Fig. 5d).

Moreover, there was no main effect of sex difference and interaction effect (Time × sex) in thermal discomfort (Average value: − 0.92 (0.72) vs − 1.19 (0.69), *p* = 0.330, and *p* = 0.193, Fig. 5e), and wetness perception in the No fan trials (Average value: − 1.60 (0.49) vs − 1.38 (0.59), *p* = 0.189, and *p* = 0.310, Fig. 5f).

## Discussion

The main findings can be drawn from this study are the females had a stronger desire for cool seeking behavior, and behavior was used to a greater extent for a given rise in body temperature (i.e., higher sensitivity) compared to males, yet the onset of cool seeking behavior was not different. The results supported our hypothesis of this study.

### The effect of sex difference on cool seeking behavior

We uniquely identified that females use cool seeking behavior to a greater extent compared to males during lower leg immersion, as evidenced by a longer cooling time, greater cumulative times of turning on a fan, and a higher slope of cool seeking behavior with rising body temperature. It is important to note that, although the cooling time is longer in females compared to males during passive heating, the T_re_, T_sk_, and thermal perceptions (including thermal sensation, thermal discomfort, and wetness perception) were not different between sexes (Figs. 2, 5). This finding indicates that females may need more cool seeking behavior to keep the same increased level of T_re_ and T_sk_ compared to males. In other words, females likely rely on cool seeking behavior to a greater extent to seemingly compensate for their lower capacity of autonomic thermoregulation to heat loss compared to males when cool seeking behavior was allowed in hyperthermic conditions, which is consistent with findings from an exercise protocol (Vargas et al. 2019b). This assumption was supported by previous studies reporting that the higher evaporation induced by a higher LSR and higher body surface area in males compared to females (Burse 1979; Yanovich et al. 2020), and our results (a higher LSR on the forearm, whole body sweat loss, and BSA in males compared to females). Thus, the females used a greater extent of cooling behavior to compensate for an attenuated potential perceived cooling effect of this heat dissipation as a lower evaporation compared to males.

Interestingly, we did not observe a sex difference in thermal discomfort despite that cooling behavioral was engaged to a greater extent and thermal discomfort is commonly reported as the precursor to engaging in thermal behaviour. The possible reason for this behavioral response may be influenced more by factors beyond the initial perception of discomfort. These can include physiological, psychological, cultural, and environmental influences that affect how individuals choose to cope with heat. Previous studies suggested that women may have a higher perceived risk of heat-related illnesses or discomfort (Gustafsod 1998; Kahan et al. 2007), and higher expectations about expressing discomfort and more acceptable to take action to cool down in specific environments (Robinson et al. 2003; Skolnick et al. 2013), which suggest that women may be more attuned to subtle changes in their thermal environment and might prioritize comfort or safety differently, whilst also being more proactive in seeking comfort due to a greater sensitivity to temperature changes or because they prioritize comfort more in certain settings (e.g., workplaces, public spaces). These differences lead them to engage in cooling behaviors more proactively, even if they report similar levels of thermal discomfort as men. For instance, women would more readily use cooling strategies like drinking water or using a fan if they perceive these actions as more socially acceptable or effective.

### The effect of sex difference on thermal perception

It has long been postulated that modifications to the structure and operation of cutaneous thermal sensors (Brück 1986) cause changes in thermal sensitivities, such as decreases in the number or size of warm spots in the skin or changes in the neurotransmission speed of nerves (Meliala et al. 1999). The studies that investigated the sex difference in thermal perception have produced equivocal results. Two previous studies (Golja et al. 2003; Gerrett et al. 2014) showed the onset of warmth perception is lower in females compared to males. However, Vargas et al. (2019) demonstrated that there is no difference in thermal perception between males and females during exercise over time, which is consistent with our results (Fig. 5), as we did not observe this sex difference in thermal perception between sex both in Fan and No fan trials in this study. The possible reason for this controversial result is that previous studies (Lan et al. 2008; Inoue et al. 2016; Filingeri et al. 2018) increased the skin temperature instead of core temperature, and the skin temperature did not change a lot in this study. It was reported that thermal sensation is dictated by skin temperature instead of core temperature (Attia 1984).

### The effect of sex difference on autonomic thermoeffectors

The significant interaction effect (Time × sex) in T_sk_, and T_b_ indicates that males and females responded differently to the 50-min lower leg immersion, with the overall pattern of change over time diverging between the two groups. Interestingly, post hoc comparisons at each individual time point did not reveal any significant differences between sexes. This suggests that while the physiological response patterns (e.g., T_sk_, and T_b_) differed over the course of the immersion, these differences were not pronounced at specific time intervals.

The onset of LSR did not differ between sexes, which concurs with previous studies (Kolka et al. 1987; Inoue et al. 2005b), which reported that there were no sex differences in the onset of sweating. However, the sensitivity of LSR was higher in males compared to females. The same tendency was also observed in the study conducted by Ogawa and Inoue (Ogawa and Sugenoya 1993; Inoue et al. 2005b), and they believe those differences were due to a smaller sweat output per sweat gland or a lower pharmacological sensitivity of the glands (Sato and Sato 1983) compared to males. The difference we explained above also led to a higher LSR on the forearm and whole body sweat loss in males. Further, it is believed that there are regional differences between the sexes in sweating, as the sex difference in heat dissipation is much greater on the forearm compared to the chest in this study (Fig. 3). Heat dissipation from the limbs is likely more effective than the trunk because the surface area-to-mass ratio of the limbs is greater than the trunk (Inoue et al. 2005b). Interestingly, these autonomic thermoeffector differences (e.g., whole body sweat loss, LSR, and SkBF) were not altered by the cool seeking behavior. However, the sex difference of sensitivity of LSR on the forearm we observed in No fan trials disappeared when the fan was allowed in Fan trials due to a longer cool-seeking behavior (Table 3).

### Consideration and limitation of this study

There are several methodological limitations that warrant discussion. Firstly, in order to account for variations in body size and the need for evaporative heat loss, the optimal procedure for identifying sex differences in thermoregulation uses a set rate of metabolic heat production proportional to body mass (Cramer and Jay 2014). We did not intentionally match groups for body surface area, percent body fat or fitness in this study. However, males and females differ in terms of body size throughout the population, thus the technique of matching for body size in laboratory experiments may lose some ecological validity. Moreover, we employed lower leg passive heating instead of exercise to attenuate the effect of fitness. A second consideration is that we did not test females in the luteal phase. Previous research indicates that the core temperature is higher in the luteal phase compared to the follicular phase (Inoue et al. 2005b). Thus, it is possible that cool-seeking behavior may be initiated to a greater extent during the luteal phase. Finally, the 7-point Olesen and Brager thermal perception measurement scales (Olesen and Brager 2004), which were employed in this investigation, might not have been sensitive enough to detect small variations in thermal perception, especially as the measurement time interval was set at every 10 min. Moreover, it was reported that when thermal perceptions were repeatedly reported, previous sensory scores can be set by the participants as reference values and the subsequent scores may be given based on the previous point of reference during exercise (Raccuglia et al. 2018). This anchoring bias might have an effect on thermal perception during lower leg immersion. Therefore, it is possible that the sex difference on thermal perception might be revealed in a circumstance of a higher air velocity, shorter time interval, and more precise measurement scale.

### Perspectives and significance

The findings from this study highlight sex-related thermal behavioral differences during passive conditions, whereby females used cool seeking behavior to a greater extent with a higher propensity to maintain a similar thermal perception. However, whether physical characteristics (e.g., body fat, VO_2max_, etc.) contribute to a greater cool seeking behavior in females and how they contribute to this result needs further investigation. Finally, this study may shed light on sex-specific cool seeking responses to heat stress, which can inform heat stress management practices in occupational settings like construction sites and industrial workplaces. For instance, employers can create cooling stations or provide more frequent breaks for female workers. Recognizing these differences allows female employers and regulatory bodies to implement targeted measures (such as adjusting work/rest cycles and hydration protocols, to prevent heat-related illnesses and injuries), and targeted air conditioning, gender-sensitive building designs, wearable cooling devices.

## Conclusions

Findings from this study revealed that females rely on cool seeking behavior to a greater extent than males even when experiencing comparable increases of body temperatures. Moreover, females might depend more on cool seeking behavior rather than autonomic thermoregulation for heat dissipation compared to males.

## Data Availability

Data will not be shared in public and will be available upon request by the readers with the contact of the corresponding author.
